# Downregulated Platelet miR-1233-5p in Patients with Alzheimer’s Pathologic Change with Mild Cognitive Impairment is Associated with Aβ-Induced Platelet Activation via P-Selectin

**DOI:** 10.3390/jcm9061642

**Published:** 2020-05-29

**Authors:** Bo Kyung Lee, Min Hee Kim, Sang Yoon Lee, Sang Joon Son, Chang Hyung Hong, Yi-Sook Jung

**Affiliations:** 1 College of Pharmacy, Ajou University, Suwon 16499, Korea; pfiffer@ajou.ac.kr (B.K.L.); or mini9943@ajou.ac.kr (M.H.K.); 2 Research Institute of Pharmaceutical Sciences and Technology, Ajou University, Suwon 16499, Korea; 3 Department of Biomedical Sciences, School of Medicine, Ajou University, Suwon 16499, Korea; sangyoon@ajou.ac.kr; 4 Department of Psychiatry, School of Medicine, Ajou University, Suwon 16499, Korea; sjsonpsy@gmail.com

**Keywords:** mild cognitive impairment, platelets, miRNA, amyloid-beta, P-selectin

## Abstract

MicroRNAs (miRNAs) have been proposed as a promising biomarker for various diseases including Alzheimer’s disease (AD). More attention has recently been focused on the diagnosis and treatment at earlier stage of mild cognitive impairment (MCI) for preventing its progression to AD. To identify potential pathologic markers for Aβ(+)MCI (Alzheimer’s pathologic change with MCI), we investigated miRNA expression profiles in the platelets from patients with Aβ(+)MCI, in comparison with those from Aβ(−)MCI (Non-Alzheimer’s pathologic change with MCI) and CNI (cognitively normal individuals). We found that let-7i-5p, miR-125a, miR-1233-5p, and miR-6787-5p were significantly downregulated, while miR-6880-5p expression was upregulated. Of these, only miR-1233-5p was significantly downregulated by Aβ treatment in both human platelets and their precursor megakaryocytes (MEG-01 cells). We explored the role of miRNAs by using miRNA mimics or inhibitors, and found that the diminished level of miR-1233-5p was associated with Aβ-induced increase in the expression of P-selectin and cell adhesion to fibronectin. Our results further indicated that Aβ-induced increase in platelet/MEG adhesion to fibronectin is likely mediated via P-selectin. In conclusion, this study suggests the downregulation of platelet-derived miR-1233-5p as a pathologic marker for Aβ(+)MCI.

## 1. Introduction

Despite the increasing number of patients with Alzheimer’s disease (AD), the development of AD therapy to target the underlying pathogenesis has been futile. This has raised questions about the current strategies employed to treat dementia. Alzheimer’s pathologic changes were shown to precede the onset of dementia and even minor cognitive deficits by 10 to 20 years [1,2]. The underlying processes driving Alzheimer’s pathology, slowly progress over time from a clinically quiet stage to mild cognitive impairment (MCI) stage and eventually AD with dementia [1]. Most damage is considered to be irreversibly accelerated upon the onset of dementia, thereby limiting the potential effect of any therapy targeting the pathogenic process [3]. Hence, the attention has been currently shifting towards treatment at earlier stages of the disease, thus delaying the progression of MCI to AD [4]. Therefore, diagnosis of patients with MCI at earlier stages of prodromal AD, is imperative. Current techniques for AD diagnostic procedures, such as neurological imaging of AD biomarkers [5,6,7] and cerebrospinal fluid (CSF) protein assays, are actually impractical for large populations because of their high cost and invasiveness. Thus, research efforts are directed towards the search for cost-effective biomarkers from less invasive biofluids such as the blood [8].

Growing evidence has demonstrated the potential role of microRNAs (miRNAs) as diagnostic biomarkers for AD [8,9]. miRNAs are small, noncoding RNA molecules comprising 18–22 nucleotides. As miRNAs play a crucial role in regulating post-transcriptional gene expression through binding to target mRNAs, alterations in their levels are recognized as a critical factor affecting cellular processes and human diseases. While the majority of miRNAs are present in cells, a significant amount is released by cells under physiological and pathological conditions into the extracellular space, including the blood and CSF in the form of circulating miRNAs [10,11]. Circulating miRNAs are highly stable owing to their small size and association with extracellular vesicles and RNA-binding proteins.

It has been reported that platelets are similar to peripheral neurons in terms of the release of neurotransmitter, such as serotonin, likely providing information about neuronal functions in AD [12]. Platelets express large amounts of amyloid precursor protein (APP). The platelet-derived APP pools constitute more than 90% of circulating APP and serve as a main source of Aβ-peptide generation in whole blood [13]. Moreover, accumulating evidence has indicated that platelets adhere to vascular amyloid plaques and are recruited to the sites of cerebral amyloid angiopathy (CAA), thereby contributing to AD-associated vascular lesions [14,15]. Platelets are also rich in miRNAs and release substantial amounts of circulating miRNAs [16]. Therefore, it is conceived that certain circulating miRNAs can play an important role in platelet-associated pathologic processes including vascular lesions. In addition, platelets have been considered as a potential biomarker because they retain higher stability than serum or CSF [17]. Although there have been extensive studies on miRNAs as AD biomarkers using hippocampus and cortex tissues and fluid samples such as serum and CSF, relatively little is known regarding platelet miRNAs as MCI markers to date.

In this study, we aimed to identify platelet-derived miRNAs as pathologic markers for Aβ(+)MCI. For this purpose, we categorized MCI patient group and cognitively normal individual (CNI) group through neuropsychological test scores, and further subcategorized the MCI patients into Aβ(+)MCI (Alzheimer’s pathologic change with MCI) and Aβ(−)MCI (Non-Alzheimer’s pathologic change with MCI) groups based on neuroimaging results from Aβ-positron emission tomography (Aβ-PET) scan. We then investigated miRNA expression profiles in the platelets from Aβ(+)MCI, Aβ(−)MCI, and CNI groups. We also evaluated the role of miRNAs by examining direct effects of Aβ on selected miRNA levels using cell models of human platelets and their precursor megakaryocytes (MEG-01). We further tested miRNA mimics or inhibitors of selected miRNA candidates for their modulating effects on Aβ-dependent platelet activation (P-selectin expression) and cell adhesion.

## 2. Materials and Methods

### 2.1. Study Population

The biospecimens and data used in this study were provided by the Biobank of Ajou University Hospital, a member of the Korea Biobank Network. The subjects who underwent diagnostic evaluation at the Department of Psychiatry, Ajou University Hospital, were asked to participate in this study. All subjects provided their informed consent before participating in the study. The study was conducted in accordance with the Declaration of Helsinki, and the protocol was approved by the Ethics Committee of Ajou University Hospital (Project No. AJIRB-BMR-KSP-18-038) and Ajou University (Project No. 201802-HM-EX-001). After the diagnostic assessment of the study population, 32 patients were classified into three groups, namely, CNI (n = 9), Aβ(−)MCI (n = 12), and Aβ(+)MCI (n = 11), according to scores of neuropsychological tests such as the Mini-Mental State Examination (MMSE), sum of Box of Clinical Dementia Rating (CDR-SB), and Global Deterioration Scale (GDS), and neuroimaging data from Aβ-PET scan and magnetic resonance imaging (MRI). Subjects from CNI group were matched to patients with Aβ(−)MCI and Aβ(+)MCI for age, education, body mass index (BMI), heart rate (HR), and use of antiplatelet medication.

### 2.2. Aβ_1-40_ Preparation

Aβ protein fragment 1-40 (Aβ_1-40_; Abcam, San Francisco, CA, USA) peptides were dissolved in ammonium hydroxide (NH4OH; 4% final volume, Sigma-Aldrich, St Louis, MO, USA) and then mixed with phosphate-buffered saline (PBS; pH 7.4, Invitrogen, Carlsbad, CA, USA) to obtain a 1 mg/mL solution. Aliquots were immediately stored at −80 °C and centrifuged for 15 min at 17,000× *g* before use to remove pre-aggregated materials [18].

### 2.3. Platelet Purification

Blood samples were centrifuged (100× *g*, 10 min, 22 °C) to obtain platelet-rich plasma (PRP). The PRP was further centrifuged at 1000× *g* for 10 min, and the platelet pellet was resuspended in Tyrode’s buffer (134 mmol/L sodium chloride [NaCl], 2.9 mmol/L potassium chloride [KCl], 0.34 mmol/L disodium phosphate [Na_2_HPO_4_], 12 mmol/L sodium bicarbonate [NaHCO_3_], 20 mmol/L HEPES, and 1 mmol/L magnesium chloride [MgCl_2_], pH 7.4). Tyrode’s buffer was warmed to 37 °C in a water bath before use; all reagents were purchased from Sigma-Aldrich (St Louis, MO, USA).

### 2.4. miRNA Microarray

Total platelet-derived RNA was extracted using the miRNeasy Serum/Plasma Advanced kit (Qiagen, Hilden, Germany) following the manufacturer’s instructions. Extracted RNA was sent to Macrogen Microarray Service (Seoul, Korea) for Affymetrix Human Gene 2.0 ST Array profiling (Affymetrix, Thermo Fisher Scientific, San Jose, CA, USA). Data were automatically extracted in Affymetrix data extraction protocol using the software provided by Affymetrix GeneChip^®^ Command Console^®^ Software (AGCC, Thermo Fisher Scientific, San Jose, CA, USA). The CEL files import, miRNA level RMA+DABG-All analysis, and result export were performed using Affymetrix^®^ Power Tools (APT) Software (Affymetrix, Thermo Fisher Scientific, San Jose, CA, USA) and the multi-average method. Array data were filtered by probes annotated species. The comparative analysis between test and control samples was performed using an independent t-test and fold change (FC), wherein the null hypothesis assumed no difference among groups. False discovery rate (FDR) was controlled by adjusting *p*-values using the Benjamini–Hochberg algorithm. All statistical tests and visualization of differentially expressed genes were conducted using R statistical language 3.3.3.

### 2.5. miRNA Real-Time Quantitative Polymerase Chain Reaction (RT-qPCR)

The expression of miRNAs was analyzed with the TaqMan miRNA RT-qPCR method (Applied Biosystems, Foster City, CA, USA) on Rotor-Gene^®^Q (Qiagen, Hilden, Germany). In brief, 10 ng total RNA was reverse transcribed using the TaqMan miRNA reverse-transcription kit (Applied Biosystems, Foster City, CA, USA) and a specific reverse-transcription stem-loop primer according to the manufacturer’s protocol. Optimum reaction conditions were determined using 5 μL of Universal Master Mix containing dNUTPs, MgCl_2_, reaction buffer, AmpliTaq Gold, 90 nM of primer(s), and 250 nM of a fluorescently labeled TaqMan probe (Applied Biosystems). Finally, 2 μL template cDNA was added to the reaction mixture. The primer/TaqMan probe combinations were designed for each target sequence. Amplifications were performed starting with a 10 min template denaturation step at 95 °C, followed by 40 cycles at 95 °C for 15 s and 60 °C for 1 min. The expression of each miRNA relative to the endogenous controls was determined using the 2^−ΔCt^ method. miRNA levels were expressed as FC in target miRNA expression relative to has-miR-RNU6B expression. All samples were amplified in triplicates, and data were analyzed using Q-Rex Software (Qiagen, Hilden, Germany).

### 2.6. Platelet Spike-in Experiment

Freshly drawn PRP samples from healthy volunteers were obtained from the Korean Red Cross Center, a blood donation facility for research purposes. The study was conducted in accordance with the Declaration of Helsinki, and the protocol was approved by the Ethics Committee of the Ajou University (Project No. 201903-HM-EX-001). The PRP was centrifuged at 1000× *g* for 10 min at 22 °C without brake to pellet platelets. The supernatant obtained was termed as platelet-poor plasma (PPP). The obtained platelet pellet was washed twice with Tyrode’s buffer (pH 7.4) and resuspended in 1/20 volume of the original PRP to obtain a 20× stock platelet solution. The 20× platelet solution was spiked back into the PPP from the same donor to achieve 200%, 100%, 50%, and 5% spike-ins. Immediately after spiking, the RNA was extracted and the level of miRNA was analyzed by RT-qPCR as previously described [19]. R value was calculated by the Pearson’s correlation coefficient.

### 2.7. Human MEG-01 Cells Transfection

MEG-01 cells were purchased from the American Type Culture Collection (ATCC, Manassas, VA, USA) and cultured in Roswell Park Memorial Institute (RPMI)-1640 medium supplemented with 10% heat-inactivated fetal bovine serum (FBS), 100 U/mL penicillin, and 100 μg/mL streptomycin (Invitrogen) at 37 °C in a 5% CO_2_ humidified atmosphere. MEG-01 cells were transfected with 100 nM miRNA mimics and an anti-miRNA oligonucleotide negative control using Lipofectamine RNAi Max (Invitrogen) according to the manufacturer’s protocol. Specific miRNA mimics, inhibitors, or negative control were purchased from Bioneer (Daejeon, Korea).

### 2.8. Flow Cytometry

Flow cytometric analyses were performed as previously described [20]. The final densities of platelets and MEG-01 cells were 1.5 × 10^8^ and 2 × 10^7^ cells/mL, respectively. Platelets and MEG-01 cells were activated with 10 μM Aβ_1-40_ for 1 h at room temperature and washed twice with Tyrode’s buffer and HEPES buffer, respectively. The cells were immediately fixed with 2% paraformaldehyde on ice for 10 min and then stained with P-selectin antibodies conjugated to PE-Cy5 (Pharmingen, San Diego, CA, USA) for 30 min at 37 °C. Surface fluorescence was assessed using a FACSAria III flow cytometer (BD Biosciences, San Jose, CA, USA).

### 2.9. Adhesion Assay with Platelets or MEG-01 Cells

For the adhesion assay, washed platelets or MEG-01 cells were added to a glass bottom dish pre-coated with fibronectin (Sigma-Aldrich) for 1 h. The cells were washed and incubated at 2.5 × 10^5^ cells/mL density for 1 h in the presence or absence of Aβ_1-40_ and an anti-human P-selectin monoclonal antibody (R&D Systems, Minneapolis, MN, USA). The supernatant was aspirated, and adherent cells were gently washed with PBS and then stained for 30 min at 37 °C with cell tracker green 5-chloromethylfluorescein diacetate (Invitrogen). For each well, five random fields were captured, and adherent cells were quantified for coverage using ImageJ.

### 2.10. Statistical Analysis

All data are expressed as mean ± standard error of mean (SEM). Two-tailed t-tests were used to examine differences in continuous variables overall and at each time point under study in different comparison groups. A value of *p* < 0.05 was considered statistically significant.

## 3. Results

### 3.1. Characteristics of the Study Population

Subjects of CNI, patients with Aβ(−)MCI and Aβ(+)MCI groups were matched with respect to all variables except for Aβ levels in brain (Aβ-PET Scan) and neuropsychological test scores. Diagnosis for cognitive function was based on patient interviews with cognitive testing by experienced neuropsychologists, whose test collection included MMSE, CDR-SB, and GDS scores. Regardless of our intention, more female patients have been recruited in the Aβ(−) group than Aβ(+) group although the reason is not clear. In the present study, MMSE, CDR-SB, and GDS were significantly changed in the Aβ(−)MCI and Aβ(+)MCI group compared to the CNI group. In the Aβ-PET scan results, Aβ levels in all regions of brain were significantly increased in Aβ(+)MCI group compared to either CNI or Aβ(−)MCI. In addition, the atrophy of right hippocampus and the apolipoprotein epsilon4 allele (ApoE ε4) carrier were shown to be significant in Aβ(+)MCI group but not in Aβ(−)MCI compared to CNI (Table 1).

### 3.2. Expression Profiles of Platelet-Derived miRNAs in Aβ(−)MCI and Aβ(+)MCI Group

To determine the expression profile of the miRNA associated with Aβ(+)MCI, we performed a miRNA array analysis for platelets from CNI, Aβ(−)MCI, and Aβ(+)MCI groups. Of more than 2000 mature miRNAs detected, five miRNAs showed an FDR-adjusted *p* < 0.05 and a > 1.5-fold change (FC) in Aβ(+)MCI group as compared to CNI group. Of these, miR-6880-5p (FC = 2.09, *p* = 0.025) was upregulated. The expression of four miRNAs, including that included let-7i-5p (FC = 4.16, *p* = 0.043), miR-125a-5p (FC = 1.70, *p* = 0.035), miR-1233-5p (FC = 1.64, *p* = 0.003), and miR-6787-5p (FC = 1.61, *p* = 0.008), was downregulated in the platelets from patients with Aβ(+)MCI compared to CNI subjects (Table 2). No significance in FC was shown for the selected five miRNAs between CNI and Aβ(−)MCI groups, suggesting that these five miRNAs may be associated with the role of Aβ in human platelets.

### 3.3. Identification of Let-7i-5p, miR-125a-5p, and miR-1233-5p as Platelet-Derived miRNAs

**Figure 1:**
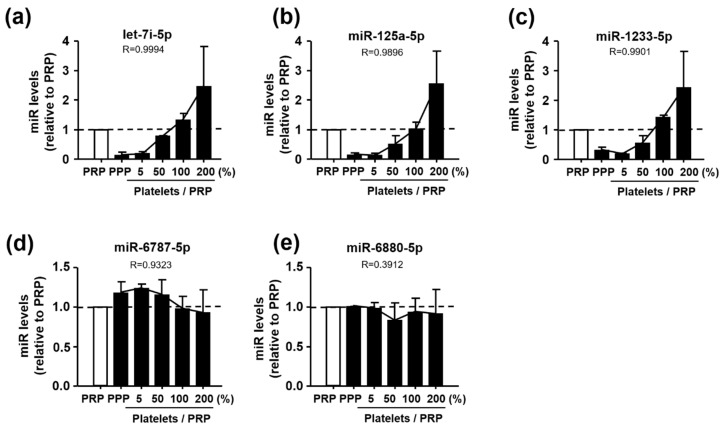
Levels of (**a**) let-7i-5p, (**b**) miR-125a-5p, (**c**) miR-1233-5p, (**d**) miR-6787-5p, and (**e**) miR-6880-5p in relation to the platelet-rich plasma (PRP) after spiking back platelets into the platelet-poor plasma (PPP) (0%–200%) in the platelet spike-in experiment. Data are mean ± SEM of seven experiments. R value was calculated by the Pearson’s correlation coefficient.

To identify the miRNAs that were derived from platelets, we isolated platelets from the PRP and spiked them back into the PPP at increasing concentrations of 5%, 50%, 100%, and 200% of the initial volume [19]. The liver-specific miR-122 was used as a control because it is a well-known miRNA unaffected by platelets. In comparison with miR-122 level, let-7i-5p, miR-125a-5p, and miR-1233-5p levels significantly increased with an increase in the concentration of platelets; however, miR-6787-5p and miR-6880-5p remained largely unchanged after the addition of platelets (Figure 1). These spike-in experiments support that let-7i-5p, miR-125a-5p, and miR-1233-5p, but not miR-6787-5p and miR-6880-5p, are originated from platelets.

### 3.4. Effects of Aβ_1-40_ on Expression Levels of Let-7i-5p, miR-125a-5p, and miR-1233-5p in Human Platelets and MEG-01 Cells

**Figure 2:**
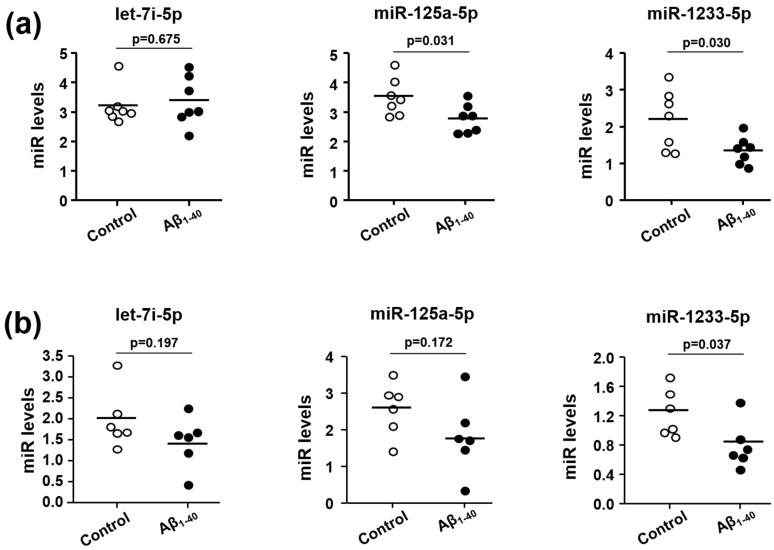
Direct effects of Aβ on let-7i-5p, miR-125a-5p, and miR-1233-5p expression in platelets (**a**) and MEG-01 cells (**b**). (**a**) Results obtained in healthy platelets (n = 7) in the absence or presence of 10 μM Aβ_1-40_ for 1 h. (**b**) Results obtained in human megakaryocytes (MEG-01 cells, n = 6) in the absence or presence of 10 μM Aβ_1-40_ for 1 h. Data are mean ± SEM.

To investigate the relevance of the three miRNAs altered in Aβ(+)MCI, we examined the direct effect of Aβ_1-40_ on the expression levels of let-7i-5p, miR-125a-5p, and miR-1233-5p using platelets from healthy volunteers. As Aβ_1-40_, the main species of circulating Aβ in plasma, is known to be released from activated human platelets and thereby stimulating platelets, we used Aβ_1-40_ for all experiments in this study [21]. miR-125a-5p and miR-1233-5p levels were significantly downregulated in the presence of Aβ_1-40_ (Figure 2a). We also investigated those miRNA levels in a well-known platelet precursor megakaryocyte cell line, MEG-01 cells, which are widely used in the field of platelet research owing to their platelet-like properties [22]. As levels of miR-125a-5p and miR-1233-5p were significantly reduced in Aβ_1-40_-stimulated platelets, we expected similar results in Aβ_1-40_-stimulated MEG-01 cells. As shown in Figure 2b, only the expression of miR-1233-5p, but not miR-125a-5p, was downregulated by Aβ_1-40_. This result suggests that the reduced level of miR-1233-5p in the presence of Aβ_1-40_ can be maintained from the precursor megakaryocyte state to mature platelet state.

### 3.5. Role of miR-1233-5p in the Activation of Platelets and MEG-01 Cells

**Figure 3:**
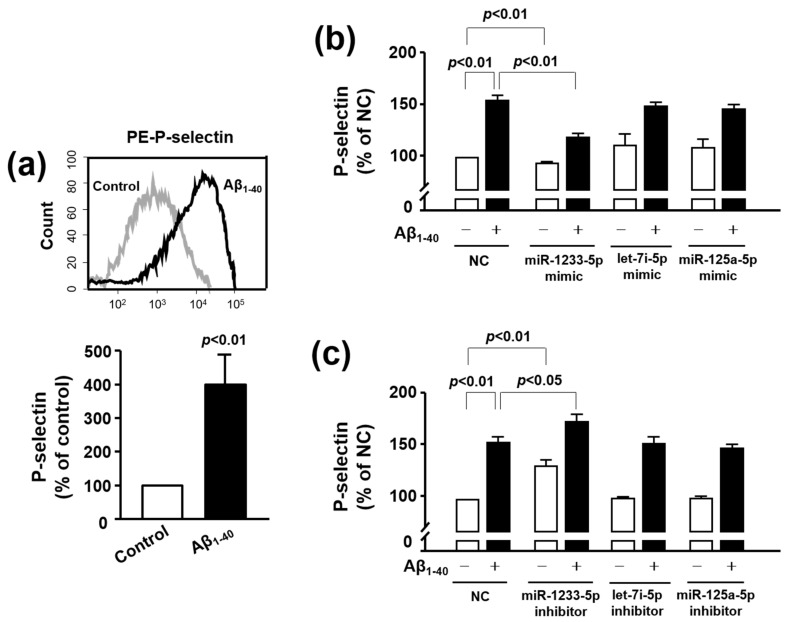
Role of let-7i-5p, miR-125a-5p, and miR-1233-5p in the Aβ_1-40_-induced P-selectin expression in platelets and MEG-01 cells. (**a**) Platelet activation status at basal level (gray curve) and after 10 μM Aβ_1-40_ stimulation (black curve) was monitored by flow cytometric measurement of PE-P-selectin expression. Data are mean ± SEM of six experiments. (b and c) Overexpression or inhibition of let-7i-5p, miR-125a-5p, and miR-1233-5p expression resulted in the alteration in the level of P-selectin in MEG-01 cells. Let-7i-5p, miR-125a-5p, and miR-1233-5p were individually overexpressed (**b**) or downregulated (**c**) using a specific mimic or inhibitor in MEG-01 cells after treatment with or without 10 μM Aβ_1-40_ for 1 h. P-selectin level was modulated in these samples as compared to that in samples treated with MEG-01 negative control (NC). Data are mean ± SEM of six experiments.

P-selectin is regarded as an essential protein responsible for platelet activation, as the cell surface P-selectin levels of platelets determine the size and the stability of platelet aggregates [23]. Thus, we evaluated the effect of Aβ_1-40_ on platelet activation by examining cell surface expression of P-selectin expression using flow cytometry [14]. As shown in Figure 3a, P-selectin expression levels in platelets were robustly enhanced after 1 h stimulation with 10 μM Aβ_1-40_ (399.68 ± 98.23%). We then tested whether alterations in let-7i-5p, miR-125a-5p, and miR-1233-5p levels by Aβ_1-40_ may be associated with its stimulatory effect on platelet activation. To examine this idea, we transfected corresponding miRNA mimics or inhibitors into MEG-01 cells as anucleated platelets were resistant to transfection. As shown in Figure 3b, the relative level of P-selectin expression was significantly enhanced in the presence of Aβ_1-40_ (153.40 ± 5.62%), compared to control (100%), and this increase was significantly attenuated by miR-1233-5p mimic (119.70 ± 3.54%), but not by let-7i-5p mimic (149.06 ± 3.04%) and miR-125a-5p mimic (146.41 ± 3.87%). In contrast to the miR-1233-5p mimic, the Aβ_1-40_-induced increase in P-selectin was further increased by miR-1233-5p inhibitor (173.01 ± 6.67%) (Figure 3c). Neither let-7i-5p inhibitor (152.37 ± 6.21%) nor miR-125a-5p inhibitor (148.18 ± 3.58%) substantially affected the P-selectin change in response to Aβ_1-40_. In addition, miR-1233-5p mimic decreased the P-selectin expression in the absence of Aβ_1-40_ (95.03 ± 1.85%), whereas miR-1233-5p inhibitor increased it (131.31 ± 5.75%). This indicates that P-selectin expression is regulated by endogenous miR-1233-5p and supports that miR-1233-5p and P-selectin may be strongly correlated. The results of Figure 3 suggest that the increased P-selectin expression by Aβ_1-40_ is mediated through Aβ_1-40_-induced downregulation of miR-1233-5p.

### 3.6. The Role of P-Selectin in Adhesion Function of Platelets and MEG-01 Cells

**Figure 4:**
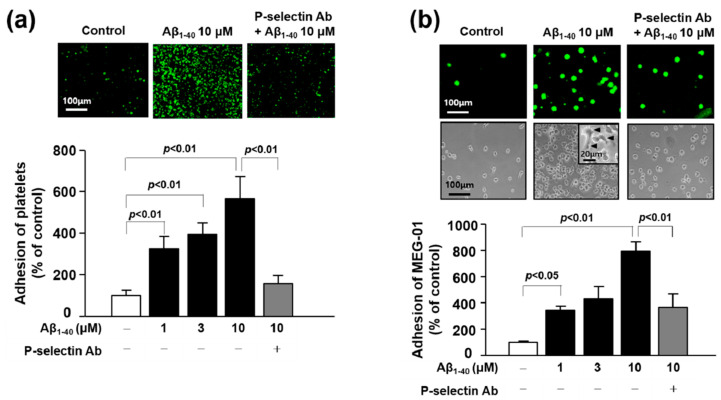
Role of P-selectin in the adhesion of platelets or MEG-01 cells. (**a**) Adhesion of platelets (green) was quantified following stimulation with 1, 3, and 10 μM Aβ_1-40_ for 1 h with or without 1 μg/mL anti-P-selectin monoclonal antibody pre-treated for 30 min on fibronectin-coated coverslips. Data are mean ± SEM of five experiments. (**b**) Adhesion of MEG-01 cells (top, green fluorescence; bottom, phase-contrast image) was quantified following stimulation with 1, 3, and 10 μM Aβ_1-40_ for 1 h with or without 1 μg/mL anti-P-selectin monoclonal antibody pre-treated for 30 min on fibronectin-coated coverslips. (insert) Arrowhead indicates spreading of MEG-01 cells. Data are mean ± SEM of six experiments.

Upon activation, platelets and MEG undergo spreading through the elongation of filopodia and lamellipodia leading to adhesion to another platelet or other cells such as monocyte, leukocytes, or endothelial cells [24,25,26]. Recent studies have revealed that these adhesion processes were significantly increased by Aβ in platelets and endothelial cells [15,20]. Consistent with these previous reports, our results showed that Aβ_1-40_ (1, 3 and 10 μM) increased platelet adhesion to fibronectin by 324.49 ± 58.26%, 392.93 ± 55.70%, and 565.53 ± 106.80%, respectively, and MEG-01 adhesion by 344.57 ± 30.54%, 431.42 ± 91.76%, and 792.81 ± 71.27%, respectively (Figure 4a,b). In addition, Aβ_1-40_ induced morphology changes with increase in adhesion of MEG-01 cells (Figure 4b insert). To determine whether the adhesion to fibronectin is mediated by P-selectin, we pretreated cells with a monoclonal anti-P-selectin antibody prior to Aβ_1-40_ treatment. As a result, Aβ-induced increase in cell adhesion was considerably inhibited by the pretreated P-selectin antibody in platelet and MEG-01 cells by approximately 72% and 54%, respectively (Figure 4a,b). These suggest that Aβ-induced cell adhesion to fibronectin is mediated via P-selectin in both platelet and MEG-01 cells.

### 3.7. Role of miR-1233-5p in the Adhesion Function of Platelets and MEG-01 Cells to Fibronectin

**Figure 5:**
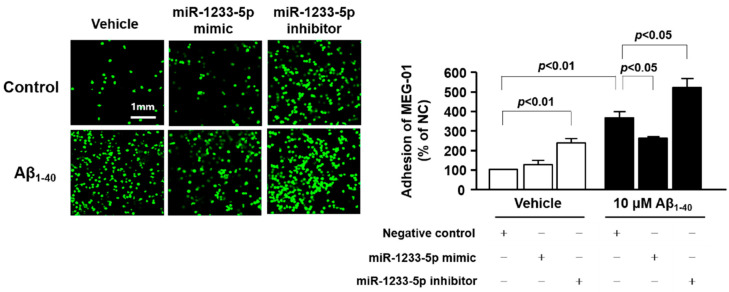
Role of miR-1233-5p in the adhesion of transfected MEG-01 cells. miR-1233-5p was overexpressed or downregulated using a specific mimic or inhibitor in MEG-01 cells for 24 h, and the cells were harvested for adhesion assay. Adhesion of transfected MEG-01 cells in the absence and presence of 10 μM Aβ_1-40_ for 1 h on fibronectin-coated coverslips was presented and quantified. Data are mean ± SEM of five experiments.

We investigated whether miR-1233-5p exerts functional effects on platelet adhesion by transfecting MEG-01 cells with a miR-1233-5p mimic or inhibitor and measuring their adhesion to fibronectin in the presence or absence of 10 μM Aβ_1-40_ for 1 h. As shown in Figure 5, the adhesion of MEG-01 cells to fibronectin was increased by 345.90 ± 27.90% after Aβ_1-40_ treatment as compared with control (100%). This effect was significantly reduced following transfection of cells with miR-1233-5p mimic (247.68 ± 7.43%) but was markedly enhanced by miR-1233-5p inhibitor (489.59 ± 42.61%), suggesting that miR-1233-5p may play an important role in the adhesion process to fibronectin. In addition, the inhibition of miR-1233-5p significantly increased adhesion of MEG-01 cells in the absence of Aβ_1-40_ (226.38 ± 19.36%). This suggests that endogenous miR-1233-5p plays an important role in fibronectin adhesion of platelet.

## 4. Discussion

In the present study, we demonstrated for the first time that the expression of miR-1233-5p was significantly downregulated in the platelets from Aβ(+)MCI patients, and that miR-1233-5p expression was directly diminished by Aβ_1-40_ in human platelets and their precursor MEG-01 cells. We further found that Aβ-induced increases in P-selectin expression, an indicative of platelet activation, and adhesion to fibronectin were significantly attenuated by transfection with miR-1233-5p mimic, and further elevated by miR-1233-5p inhibitor, suggesting a causative relationship between the downregulated miR-1233-5p and platelet/MEG activation. Taken together, our findings suggest a potential role of downregulated miR-1233-5p as a platelet-associated pathologic marker for Aβ(+)MCI.

AD is an irreversible brain disease with a gradual decline in multiple cognitive processes, leading to the need for full-time care, while MCI is regarded as a transitional state with a slight memory decline between normal aging and AD. MCI is becoming increasingly common with aging and is associated with an increased risk of progression to AD, suggesting that pathologic changes proceed at MCI stage. According to longitudinal studies, people with MCI were more likely to progress to AD than those with normal cognition [27,28], and early diagnosis followed by immediate treatment while maintaining patient’s consciousness may be more efficacious than administering medication later on [29]. Thus, it is meaningful to identify the precedent pathologic biomarkers at the MCI stage prior to the appearance of dementia symptoms and irreversible damage. Many approaches have been directed to discover early diagnostic biomarker for MCI using MRI and Aβ-PET imaging [30]. Previous studies have shown that β-amyloidosis in amnestic MCI is a predictive biomarker for progression to dementia although wide variations have been reported between β-amyloidosis in amnestic MCI and risk for dementia. Consistent with these results, a recent study on Korean patients with MCI showed that Aβ-PET(+) subjects with MCI are significantly more likely to convert to AD than Aβ-PET(−) patients [31]. These authors showed that faster converters had higher PIB retention levels at baseline than slower converters. Despite these valuable results, the brain imaging approach employed in their study is not common because of the requirement for expensive equipment, highly trained staff, and high usage cost. In this context, our study focused on patients with MCI with or without Alzheimer’s pathologic change and aimed to identify a novel marker using less invasive samples other than the brain. To the best of our knowledge, this is the first report to explore the potential of platelet-derived pathologic markers for Aβ(+)MCI.

An ideal biomarker should meet many criteria, including specificity, sensitivity, cost efficiency, and safety. However, currently available biomarkers for AD have low specificity and sensitivity. Recently, miRNAs are recognized as novel diagnostic and prognostic biomarkers with high specificity, sensitivity and stability [32]. More than 2,000 miRNAs have been discovered in human cells and many of them are specific to, or overexpressed in, certain organs, tissues, and cells [33]. Increasing studies have demonstrated the pivotal roles played by miRNAs in a wide range of physiological processes, and their deficiencies have been related to a number of diseases including AD, Parkinson’s disease, and stroke [34,35]. Several hippocampal miRNAs, including miR-370, miR-328, miR-15a, miR-138, and miR-132, have been identified as diagnostic markers for AD [36,37]. As miRNAs can be secreted or excreted into the extracellular space [38] and are detectable in plasma and serum, such circulating miRNAs have emerged as minimally invasive biomarkers. Indeed, various serum miRNAs, including miR-342-3p, miR-195, miR-155, miR-9, miR-206, and miR-29, are recently suggested as promising diagnostic markers for AD [39,40]. Nevertheless, it has also been recognized that serum/plasma profiles for AD-related markers are difficult to reproduce [41] because factors such as accompanying inflammation are likely to influence the plasma levels of these markers.

Platelets share biochemical characteristics with neurons, and are considered to be the most accessible neuron-like cells. As platelet-derived APP is also processed via identical amyloidogenic and non-amyloidogenic pathways as brain APP, they are likely to be associated with pathologic changes in dementia [21]. Platelets contain several miRNAs, mRNAs and proteins that are involved in miRNA processing and are derived from megakaryocytes [42]. Platelets release miRNA-containing microvesicles that can be transferred into other neighboring cells, such as endothelial cells, smooth muscle cells, and other blood cells, wherein they subsequently regulate protein synthesis and functions [43]. Therefore, some platelet-derived miRNAs may play a potential role in platelet-associated pathologic processes, including vascular lesions. In a similar context, recent studies have reported enhanced platelet activation and aggregation in MCI in correlation with AD progression, and an ameliorating effect of anti-platelet drugs on cognitive decline [44,45]. Based on these results, we hypothesized that some miRNAs in platelets are important in the process of pathologic changes in Aβ(+)MCI patients that are known to progress to AD faster than Aβ(−)MCI patients. In this study, we performed analysis of miRNA expression in platelets from Aβ(−)MCI and Aβ(+)MCI patients in comparison with those from CNI. With this approach, we were able to identify five platelet-derived miRNAs (let-7i-5p, miR-125a-5p, miR-1233-5P, miR-6787-5p, and miR-6880-5p) with differential expression patterns between Aβ(+)MCI and CNI, but not between Aβ(−)MCI and CNI groups. Platelet spike-in experiment results indicated that platelets were the major sources of three miRNAs (let-7i-5p, miR-125a-5p, and miR-1233-5p) (Figure 1). In the subsequent study to investigate the direct effect of Aβ on miRNA expression using human platelets and their precursor MEG-01 cells, our results showed that Aβ treatment significantly diminished miR-125a-5p and miR-1233-5p in platelets and only miR-1233-5p in MEG-01 cells. These results suggest that miR-1233-5p expression is downregulated by Aβ in both mature platelets and their precursor cells, although underlying mechanism for Aβ-induced downregulation of miR-1233-5p remains to be further clarified. We also investigated a possible causal relationship between the diminished miR-1233-5p and platelet activation in response to Aβ treatment using miRNA mimics or inhibitors using MEG-01 cells. Our current results demonstrated that the cell surface expression levels of P-selectin were significantly reduced by miR-1233-5p mimic in MEG-01 cells and, conversely, elevated by miR-1233-5p inhibitor. As the elevated expression of surface P-selectin is indicative of platelet activation, these results suggest a suppressive role of miR-1233-5p in platelet activation. P-selectin is a well-known adhesion molecule that is constitutively synthesized and packaged into storage granules in platelets, MEG and endothelial cells [46]. In general, various secretagogues, such as thrombin, histamine, and Aβ, rapidly mobilize P-selectin from storage granules to the plasma membrane, leading to platelet activation and aggregation [20,47]. It is well-known that Aβ aggregation occurs in the brain or cerebral blood vessel of AD patients. Especially, platelets contribute to Aβ aggregation in cerebral blood vessel and adhere to vascular lesion together with Aβ, resulting in cerebral amyloid angiopathy (CAA). CAA plays an important role in the severity of AD pathology because it induces degeneration or even destruction of the vessel wall and affects cerebral blood flow [14,15]. Therefore, adhesion of platelet to fibronectin by Aβ may be associated with the pathogenesis of CAA, but further studies are needed to clarify this. Consistent with these, our results showed that Aβ stimulation of platelets induced a rapid increase in cell surface expression of P-selectin. The lectin domains of P-selectins bind to each other to stabilize interactions between adjacent platelets, thereby allowing large platelet aggregate formation [23]. P-selectin mediates platelet-monocyte complex formation through promoting platelet adhesion to monocytes, which further induces subsequent adhesion of endothelial cells via intercellular adhesion molecule-1 and fibronectin [24]. It is known that the association of P-selectin with fibronectin is involved in the initial step of platelet-platelet adhesion, leading to assembled fibrillary structures [24,48]. This study notably showed that the Aβ-enhanced adhesions of platelets or MEG-01 cells to fibronectin were significantly abolished by anti-P-selectin antibody, suggesting a pivotal role of P-selectin in Aβ-induced platelet adhesion to fibronectin. In addition, bioinformatic analysis of this study have shown that a number of cell adhesion-associated genes, such as ALCAM and CADM1, are potential target genes of miR-1233-5p (Supplementary Figure S1). Overall, our results suggest that downregulated miR-1233-5p may play a role in the Aβ-induced pathologic processes including platelet adhesion. However, further study remains to be investigated to support this possibility since there is little information on the role of miR-1233-5p except for a previous study of its role in renal carcinoma [49].

In conclusion, the present study represents the first identification of diminished expression of platelet miR-1233-5p in patients with Aβ(+)MCI as a pathologic risk factor for platelet activation. This study also highlights P-selectin as a novel downstream modulator of miR-1233-5p in platelets, with a role for Aβ-induced platelet adhesion to fibronectin. Despite the high level of accuracy in the diagnosis of the patient population based on highly advanced imaging techniques such as Aβ-PET and MRI, as well as neuropsychological tests, this study has a few limitations including the small population size. Hence, further studies are warranted to investigate the diagnostic or prognostic value of miR-1233-5p in a prospective study with larger number of patients.

## Figures and Tables

**Table 1:** Demographic and clinical characteristics of patients with Aβ(−)MCI and Aβ(+)MCI.

Variable	CNI	Aβ(−)MCI	Aβ(+)MCI	*p*-Value	
				CNI vs. Aβ(−)MCI	CNI vs. Aβ(+)MCI
**Total, n**	**9**	**12**	**11**	-	-
Age (years)	73.2 ± 1.43	74.2 ± 5.67	74.6 ± 2.48	0.681	0.660
Female (%)	66.7 ± 0.17	91.7 ± 0.29	72.7 ± 0.14	0.164	0.783
Education (years)	9.78 ± 1.40	9.50 ± 5.20	9.46 ± 1.27	0.897	0.870
BMI (kg/m^2^)	25.2 ± 1.23	23.7 ± 3.28	23.1 ± 0.49	0.335	0.109
HR	74.6 ± 4.98	76.8 ± 6.45	74.2 ± 3.13	0.640	0.949
MMSE	27.3 ± 0.29	24.5 ± 3.03	24.0 ± 0.53	0.014 ^†^	< 0.001 ^†^
CDR-SB	1.22 ± 0.21	1.83 ± 0.69	2.41 ± 0.27	0.049 ^†^	0.004 ^†^
GDS	2.11 ± 0.11	2.75 ± 0.45	2.73 ± 0.14	0.002 ^†^	0.003 ^†^
Platelet Count (10^8^/mL)	2.06 ± 0.12	2.29 ± 0.93	2.33 ± 0.28	0.483	0.450
Amyloid PET (SUVr)					
Cingulate gyrus (L + R)	0.72 ± 0.02	0.69 ± 0.05	0.92 ± 0.03	0.254	< 0.001 ^†^
Frontal lobe (L + R)	0.58 ± 0.01	0.56 ± 0.05	0.79 ± 0.03	0.245	< 0.001 ^†^
Occipital lobe (L + R)	0.61 ± 0.02	0.60 ± 0.04	0.68 ± 0.01	0.535	0.009 ^†^
Parietal lobe (L + R)	0.58 ± 0.01	0.56 ± 0.04	0.79 ± 0.03	0.255	< 0.001 ^†^
Temporal lobe (L + R)	0.60 ± 0.01	0.58 ± 0.04	0.78 ± 0.04	0.226	< 0.001 ^†^
Global (L + R)	0.59 ± 0.01	0.57 ± 0.04	0.76 ± 0.02	0.281	< 0.001 ^†^
ApoE ε4 carrier (%)	0.00 ± 0.00	8.00 ± 0.28	54.4 ± 0.15	0.400	0.006 ^†^
Fazekas Scale (WMH)	1.33 ± 0.20	1.50 ± 0.52	1.27 ± 0.18	0.470	0.783
VBM					
Hippocampus (L)	0.40 ± 0.05	0.36 ± 0.05	0.37 ± 0.03	0.062	0.066
Hippocampus (R)	0.40 ± 0.03	0.36 ± 0.05	0.36 ± 0.02	0.063	0.018 ^†^

Data are expressed as mean ± SEM (range). For statistical analysis, unpaired Student’s t-test was used. ^†^ *p* < 0.05: MCI group versus CNI group. CNI, cognitive normal individual; Aβ(−)MCI, Non-Alzheimer’s pathologic change with MCI; and Aβ(+)MCI, Alzheimer’s pathologic change with MCI.

**Table 2:** Fold changes in the five most significantly detected miRNAs in the platelets from patients with Aβ(−)MCI and Aβ(+)MCI compared to those of the CNI group.

miRNA	Aβ(−)MCI		Aβ(+)MCI	
	FC	*p*-value	FC	*p*-value
hsa-let-7i-5p	−1.39	0.516	−4.16	0.043 ^†^
hsa-miR-125a-5p	1.04	0.946	−1.70	0.035 ^†^
hsa-miR-1233-5p	−1.25	0.193	−1.64	0.003 ^†^
hsa-miR-6787-5p	−1.18	0.448	−1.61	0.008 ^†^
hsa-miR-6880	1.42	0.144	2.09	0.025 ^†^

Data are expressed as fold change (FC), and false discovery rate (FDR) was controlled by adjusting the *p* value using the Benjamini–Hochberg algorithm. ^†^ *p* < 0.05: MCI group versus CNI group. CNI, cognitive normal individual; Aβ(−)MCI, Non-Alzheimer’s pathologic change with MCI; and Aβ(+)MCI, Alzheimer’s pathologic change with MCI.

## Supplementary Materials

The following are available online at https://www.mdpi.com/2077-0383/9/6/1642/s1, Figure S1 Kyoto Encyclopedia of Genes and Genomes (KEGG) pathway enrichment analysis for miR-1233-5p target genes.
