# Supplementary material for: Downregulated Platelet miR-1233-5p in Patients with Alzheimer’s Pathologic Change with Mild Cognitive Impairment is Associated with Aβ-Induced Platelet Activation via P-Selectin
Source: J Clin Med. 2020 May 29;9(6):1642. doi: 10.3390/jcm9061642 (PMC7357133; doi:10.3390/jcm9061642)
Supplement: Supplementary file 1 [file jcm-09-01642-s001.pdf]

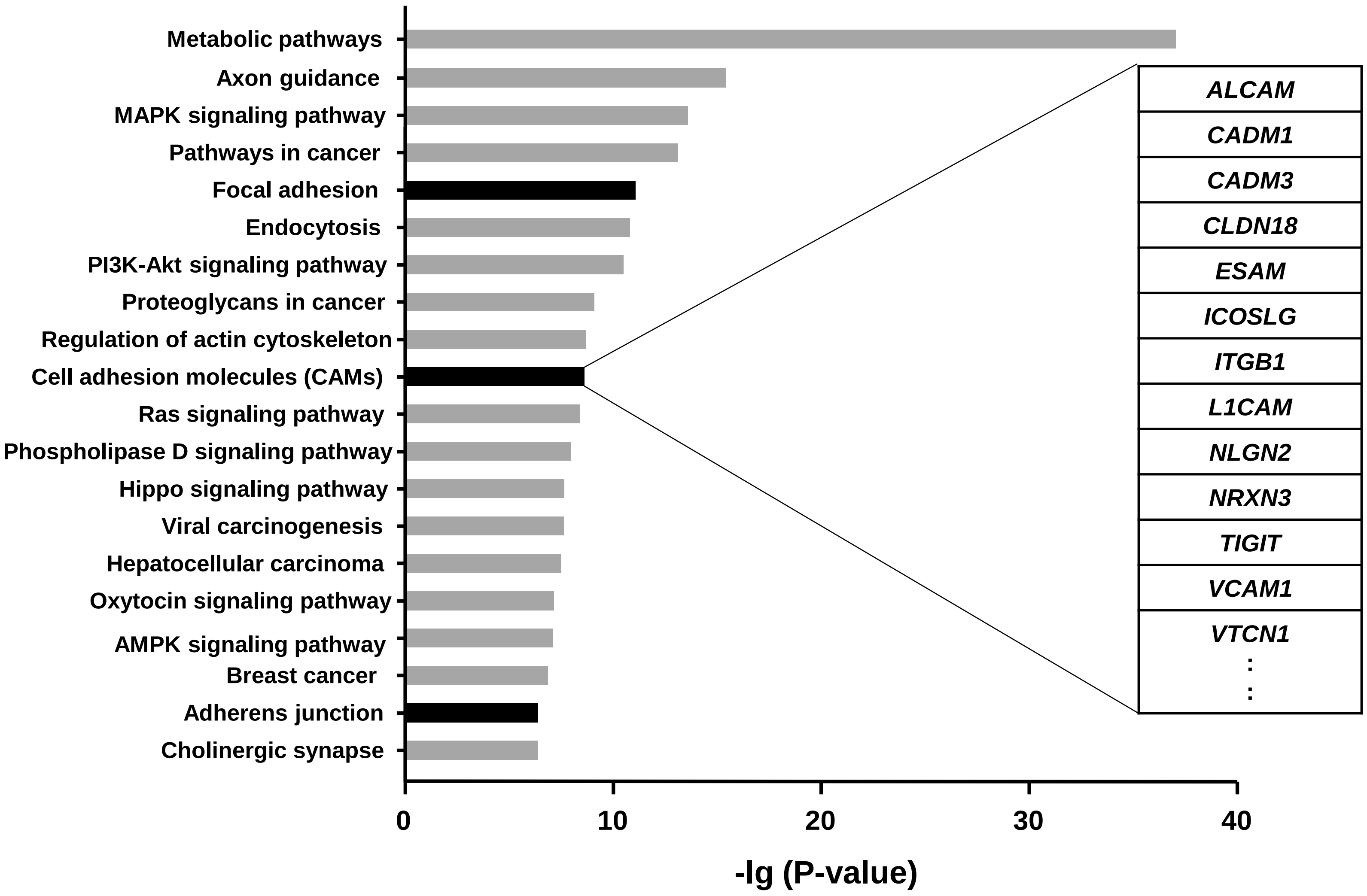

Supplementary Figure S1. Kyoto Encyclopedia of Genes and Genomes (KEGG) pathway enrichment analysis for miR-1233-5p target genes using the DAVID bioinformatics tool. The top 20 highly enriched KEGG pathways and cell adhesion molecules (CAMs)-related genes were represented.
